# Enhanced photo-fenton and photoelectrochemical activities in nitrogen doped brownmillerite KBiFe_2_O_5_

**DOI:** 10.1038/s41598-022-08966-8

**Published:** 2022-03-24

**Authors:** Durga Sankar Vavilapalli, Santosh Behara, Raja Gopal Peri, Tiju Thomas, B. Muthuraaman, M. S. Ramachandra Rao, Shubra Singh

**Affiliations:** 1 Crystal Growth Centre, Alagappa College of Technology, Anna University, Chennai, 600025 India; 2 Department of Metallurgical and Materials Engineering, Indian Institute of Technology Madras, Chennai, 600036 India; 3 Department of Energy, University of Madras, Chennai, 600025 India; 4 Nano Functional Materials Technology Centre, Department of Physics, Indian Institute of Technology Madras, Chennai, 600036 India

**Keywords:** Materials science, Nanoscience and technology, Energy science and technology, Energy harvesting, Renewable energy

## Abstract

Visible-light-driven photo-fenton-like catalytic activity and photoelectrochemical (PEC) performance of nitrogen-doped brownmillerite KBiFe_2_O_5_ (KBFO) are investigated. The effective optical bandgap of KBFO reduces from 1.67 to 1.60 eV post N-doping, enabling both enhancement of visible light absorption and photoactivity. The photo-fenton activity of KBFO and N-doped KBFO samples were analysed by degrading effluents like Methylene Blue (MB), Bisphenol-A (BPA) and antibiotics such as Norfloxacin (NOX) and Doxycycline (DOX). 20 mmol of Nitrogen-doped KBFO (20N-KBFO) exhibits enhanced catalytic activity while degrading MB. 20N-KBFO sample is further tested for degradation of Bisphenol-A and antibiotics in the presence of H_2_O_2_ and chelating agent L-cysteine. Under optimum conditions, MB, BPA, and NOX, and DOX are degraded by 99.5% (0.042 min^-1^), 83% (0.016 min^-1^), 72% (0.011 min^-1^) and 95% (0.026 min^-1^) of its initial concentration respectively. Photocurrent density of 20N-KBFO improves to 8.83 mA/cm^2^ from 4.31 mA/cm^2^ for pure KBFO. Photocatalytic and photoelectrochemical (PEC) properties of N-doped KBFO make it a promising candidate for energy and environmental applications.

## Introduction

Contaminants like organic dyes, synthetic compounds and antibiotics in wastewater are severe threat to environment and human health^1–3^. Several organic dyes have been used as a human and veterinary medicine for some of therapeutic and diagnostic procedures^4,5^. However, traces of dyes in water bodies is hazardous to environment and difficult to degrade using conventional water treatment methods due to aromatic structures, hydrophilic nature and high stability against light, and temperature etc^6^. Another organic effluent Bisphenol-A [2,2-bis (4-hydroxyphenyl) propane] or BPA widely found in wastewaters, is a raw material for manufacturing epoxy and polycarbonate plastics. Recent studies reveal that BPA has severe effects on the human health. , effects reproductive systems and causes fertility problems^7,8^. It is one of the emerging pollutants, contaminating water bodies in recent times due to excessive plastic usage. This synthetic compound is difficult to degrade in natural conditions due to its complex structure. Various techniques such as physical adsorption, biodegradation and other chemical remediation are tested for degradation of BPA, which are expensive as well as take longer time to degrade^9^. Hence, economical and energy efficient strategies are required to treat these kinds of effluents. Water pollutants like pharmaceutically active compounds such as antibiotics are also being extensively used in recent times for the treatment of infectious diseases and for enhancing agricultural production^10,11^. Their extensive use, incomplete biodegradability, partial removal using conventional water treatment plants lead to environmental contamination. Some of such antibiotics are Norfloxacin (NOX) and Doxycycline (DOX). Norfloxacin is a Fluoroquinolone antibiotic widely used for respiratory and bacterial infections^12^. Doxycycline is one of the widely used antibiotic, which is used to treat some of the most hazardous diseases such as plague and anthrax^13^. These fluoroquinolone and Doxycycline antibiotics are widely detected in surface water and other environmental matrixes due to incomplete treatment of these antibiotics in water treatment plants. A prolonged exposure to these antibiotics in aquatic environment can lead to antibiotic resistance^14,15^. As a result, pathogens become increasingly resistant to the drugs and hence it is a severe threat to the both aquatic and terrestrial organisms. These toxic, non-biodegradable pollutants are difficult to degrade/mineralize under natural conditions. Since last two decades many physical, chemical and biological techniques which have been developed to degrade/remove these contaminants from wastewater have disadvantages like high cost, longer time of degradation, and other pollutant parameters. Among various advanced oxidation processes (AOPs), photocatalytic and photo-fenton-like catalytic processes have attracted remarkable attention for the decomposition of organic effluents and antibiotics in efficient ways, the processes being both economically feasible and energy efficient.

The photocatalytic process involves redox reactions initiated by electron(e^−^)-hole (h^+^) pairs (generated by catalyst under light irradiation)^16^ leading to the formation of active species. These active species are responsible for degradation of pollutants^17^. The photo-fenton-like catalytic process is a conventional fenton process in presence of light irradiation. In fenton process, •OH radicals can be generated by reaction between Fe-based catalysts (Fe_3_O_4_, BiFeO_3_) and fenton reagent (eg: H_2_O_2_). The additional light irradiation on fenton-process leads to generation of more •OH radicals^18^. The synergistic effect between the photocatalysis and fenton reactions enhances the photodegradation of effluents^19–21^. Fe-based visible light active photocatalysts could be promising candidates for photo-fenton-like catalytic processes, which can absorb 45–50% of sunlight from entire solar spectrum, whereas ultraviolet (UV) light active photocatalysts absorbs only 3–5% of sunlight^22^. Hence it is necessary to develop visible light-driven photo-fenton-like catalysts for wastewater treatment applications.

Perovskite BiFeO_3_(BFO) is one of the well-known multifunctional material, which has a wide range of applications due to its promising magnetic, electrical and optical properties. In recent times BFO and its composites have been widely explored as photocatalyst for water splitting and wastewater treatment as well^23–28^. The bandgap of BFO (2.1–2.6 eV) falls under visible range of solar spectrum and has a theoretical photo conversion efficiency about 7%^29,30^. If the bandgap can be reduced further, it is expected that the efficiency can be enhanced improving the photodegradation performance of catalyst. Another strategy to improve the catalytic activity is creation of substantial oxygen vacancies in perovskite structures, acting as active sites for catalytic activity^31–33^. In this regard, materials with a combination of low bandgap and oxygen deficiency, such as, oxygen deficient perovskite structured/brownmillerites can be explored^34,35^. Brownmillerite oxides such as Ca_2_Fe_2_O_5_, Ca_2_Mn_2_O_5_ and Sr_2_Fe_2_O_5_ show better catalytic activity over perovskite compounds due to substantial oxygen vacancies in their structure^36–39^. KBiFe_2_O_5_ (KBFO) is one such recent brownmillerite compound which has smaller bandgap than BFO and showed promising photocatalytic activity to degrade organic effluents^40^. Nitrogen doping in KBFO can further enhance the photo-fenton activity due to presence of Fe-N_x_ active sites and reduced bandgap over bare KBFO.

Recent studies have revealed that addition of chelating agent L-Cysteine to Fe-based catalysts Fe_3_O_4_ and BiFeO_3_ enhances the catalytic activity^41,42^. L-Cysteine is a sulfur-containing amino acid with three functional groups (-SH, -NH_2_, and -COOH)^42^. Reaction of L-Cysteine with O_2_^-^ is reported to generate H_2_O_2_, which acts as the fenton reagent. Hence, N-KBFO/H_2_O_2_/L-Cysteine system could well be proposed as a promising candidate for efficient photo-fenton activity and decomposition of organic effluents and antibiotics^41,42^.

In this work N-KBFO with various N-doping concentrations has been synthesized by sol–gel method. The structural, morphology, optical properties of as prepared samples were analyzed and detailed photo-fenton activity of N-KBFO in the presence of L-Cysteine and H_2_O_2_ were investigated by degrading organic effluents Methylene blue (MB), Bisphenol-A (BPA) and antibiotics Norfloxacin (NOX) and Doxycycline (DOX) under visible light. The active species responsible for degradation of organic effluents are investigated using active species trapping experiment. The photoactivity of this N-KBFO and KBFO was also demonstrated using photoelectrochemical studies.

## Experimental section

### Preparation of N-KBFO

N-KBFO compound was prepared by conventional sol–gel technique. KNO_3_(0.1 M), Bi(NO_3_)_3_.5H_2_O (0.1 M) and Fe(NO_3_)_3_.9H_2_O (0.2 M) are taken as precursors and dissolved in 50 ml of Ethylene glycol under vigorous stirring. Here, Melamine (C_3_H_6_N_6_) was used as the source to incorporate Nitrogen. Various concentrations of melamine are dissolved in 50 ml of precursor solution, (N concentration in the precursor solution: 0, 5, 10, 15, 20, 25 and 30 mmol). After 10 h of constant stirring, the suspension was dried in an oven for 24 h at 100 °C. Finally, the dry mass was calcined at 700 °C for 6 h in a furnace^40,42^. The obtained brownish powder was labeled as KBFO, 5N-KBFO, 10N-KBFO, 15N-KBFO, 20N-KBFO, 25N-KBFO and 30N-KBFO based on its respective nitrogen doping concentration. The N-doping concentrations of KBFO, 5N-KBFO, 10N-KBFO, 15N-KBFO, 20N-KBFO, 25N-KBFO and 30N-KBFO were estimated to be 0, 0.81, 1.63, 2.16, 2.75, 3.12 and 3.63 At. % using energy dispersive X-ray spectroscopy (EDS).

### Characterization

The structural, morphological, optical, and spectroscopic studies on as prepared samples have been performed by an X-ray diffractometer (Bruker S4 pioneer) and high resolution scanning electron microscope (FESEM, TESCAN-MIRA3), UV − visible spectrophotometer (JASCO, V-730), and an X-ray photoelectron spectroscopy (SPECS GmbH, Germany) respectively.

### Photocatalytic degradation of MB, BPA, NOX and DOX

Photo-fenton reaction experiments were performed to evaluate the photocatalytic performance of as synthesized N-doped KBFO by degrading organic effluents Methylene blue MB (20 ppm), BPA (30 ppm) and antibiotics such as Norfloxacin(NOX, 30 ppm)and Doxycycline (DOX, 30 ppm) under visible light (100 mW/cm^2^, AM1.5, Xenon lamp at 25 °C). Initially aqueous MB dye solution was loaded with 50 mg/L of as synthesized catalysts and photodegradation experiments were performed to find an optimum N doping for better performance. Photo-fenton reactions were then performed by adding optimum content of H_2_O_2_ and organic ligand (L-cysteine) to effluent-catalyst suspension. Prior to light exposure, the catalyst-effluent solutions were ultrasonicated under dark for 20 min to ensure adsorption–desorption equilibrium. Then the catalyst loaded effluent suspension was placed under light and samples were collected at regular time intervals and filtered (using 0.25 µm syringe filter). The residual concentration of effluents was measured using UV–Visible spectrometer. These experiments are repeated to degrade BPA (30 ppm), antibiotics Norfloxacin (NOX, 30 ppm) and Doxycycline (DOX, 30 ppm). The percentage of degradation and first order kinetics are measured using the following expressions (Eqs. 1 and 2)

1 $$Percentage\ of\ Degradation \left( {\% D} \right) = \left[ {\frac{{C_{0} - C}}{{C_{0} }}} \right]$$

2 $$\ln \frac{{C_{0} }}{C} = kt$$

where *C*_*0*_ and *C* are the concentrations of effluent at 0 min and at time interval t respectively. k is the degradation rate constant.

### Photoelectrochemical studies

Photoelectrochemical (PEC) studies were carried out using Electrochemical workstation (AUTOLAB, PGSTAT 204 FRA32M) under illumination of 100 mW/cm^2^ (1 Sun) of light intensity using a 150 W Tungsten-halogen lamp source. KBFO and N doped KBFO electrodes are prepared by coating slurry of active material on FTO (a mixture of α-terpineol and ethyl cellulose mixture used as binder). PEC performance was investigated through linear sweep voltammetry (LSV), chronoamperometry (CA) and Electrochemical impedance spectroscopy (EIS) studies.

### DFT calculations

In order to estimate the theoretical bandgap of KBFO and N-doped KBFO Density functional theory (DFT) calculations were conducted. DFT calculations are performed using the ultrasoft pseudopotential (USPP) method in the Quantum ESPRESSO package^43^. The exchange correlation energy is approximated using the Perdew-Burke-Ernzerhof (PBE) generalized gradient approximation (GGA) functional^44^. We have used the plane wave energy cutoff of 130 Ry and a 4 $\times$ 4 $\times$ 4 Monkhorst–Pack grid^45^. Self-consistency in calculations is achieved until the total energies have converged to 10^–6^ eV/cell, and the structures have been relaxed until the Hellman-Feymann forces relaxed to less than 10^–2^ eV/Å. The electronic structure is calculated by sampling the Brillouin zone with a set of high symmetry k-points^46^.

## Results and discussions

**Figure 1:**
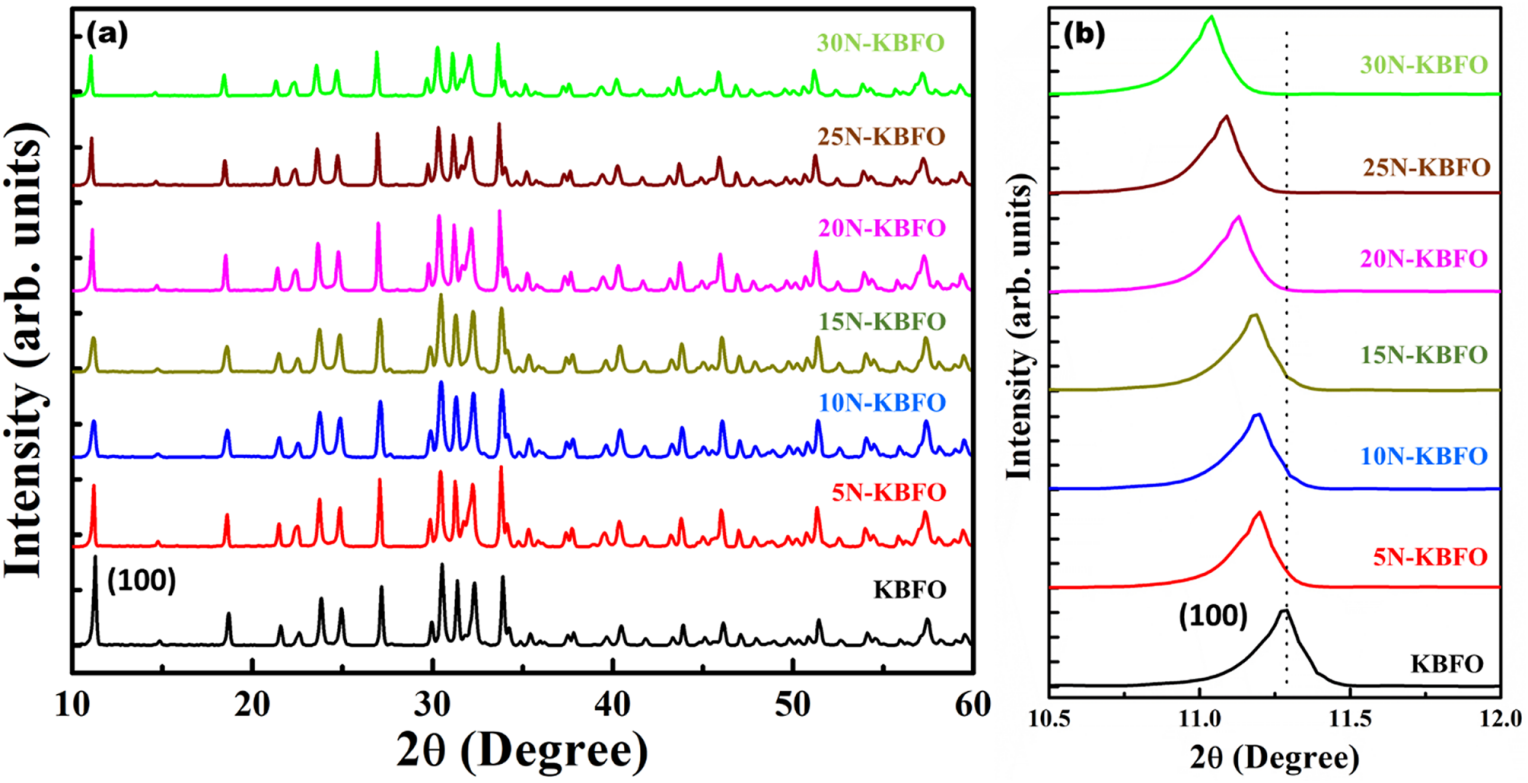
(**a**) XRD patterns for KBFO with various N-doping concentrations (**b**) Magnified view of (100) plane in XRD showing a shift towards lower 2θ.

**Figure 2:**
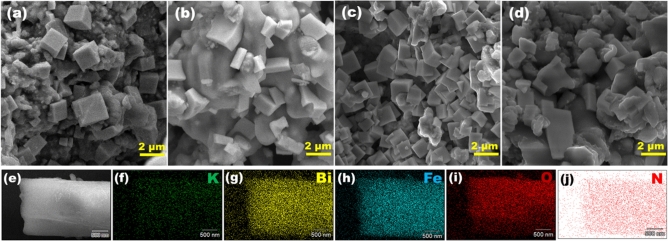
SEM images corresponding to (**a**) KBFO (**b**) 10N-KBFO (**c**) 20N-KBFO (**d**) 30N-KBFO. (**e**–**j**) EDS mapping images of 20N-KBFO sample showing uniform distribution of K, Bi, Fe, O and N elements.

Figure 1a shows the XRD pattern of N-KBFO with various nitrogen doping concentrations. The XRD pattern is in good agreement with monoclinic structure (P2/c) of KBFO^40^. No impurity phases are observed after N-doping for all the concentrations. The diffraction peaks shift towards lower 2θ values with increase in N-doping concentration, due to the substitution of lower ionic radius N-atom in O-site. The shift in lattice plane (100) corresponds to N-doping concentration shown in Fig. 1b. A similar trend was observed in earlier reports on nitrogen doped metal-oxide systems^47,48^. Figure 2a–d shows the SEM images of KBFO, 10N-KBFO, 20N-KBFO and 30N-KBFO respectively. All samples exhibit randomly oriented rectangular like grains. The EDS elemental mapping (Figure(e-j)) of 20N-KBFO confirms the uniform distribution of constituent elements throughout the sample and presence of N is observed in N-doped sample.

**Figure 3:**
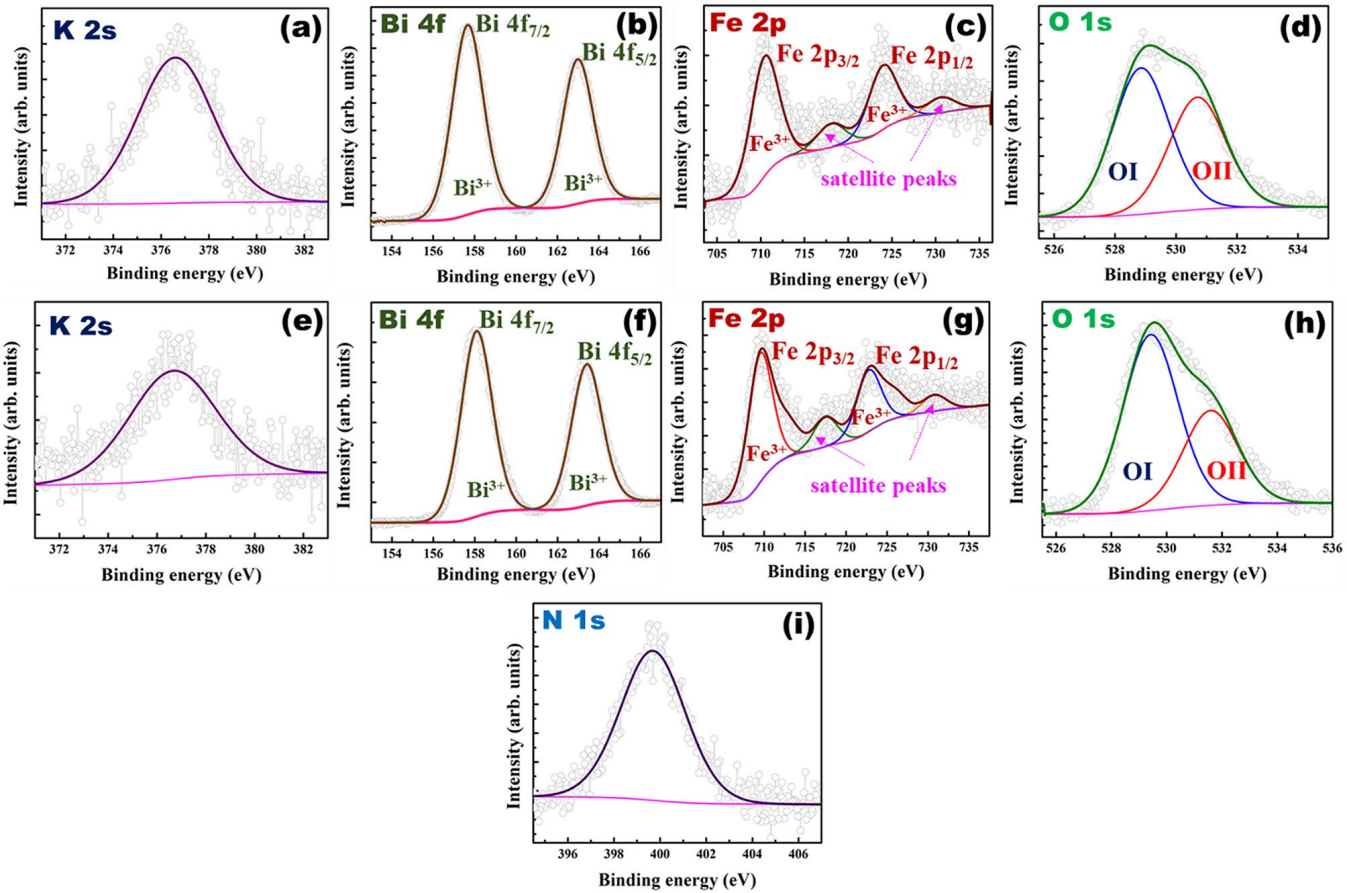
X-ray photoelectron spectra and the corresponding fits belonging to KBFO and 20 N-KBFO samples: (**a**–**d**) K 2s, Bi 4f, Fe 2p, and O 1s corresponding to KBFO (**e**–**i**) K 2s, Bi 4f, Fe 2p, O 1s and N 1s corresponding to 20N-KBFO.

Valence states of constituent elements as well as presence of nitrogen in N doped KBFO were confirmed by XPS analysis [Fig. 3]. The XPS spectra of K 2 s peak lies at 376.60 eV and 376.74 eV for pure KBFO and 20N-KBFO respectively as shown in Fig. 3a&e^40^. XPS spectra corresponding to Bi 4f. for both KBFO and 20N-KBFO samples split into two spin orbit peaks [Fig. 3(b&f)], occurring at 157.6 ± 0.5 eV and 163.1 ± 0.5 eV and ascribed to Bi 4f_7/2_ and Bi 4f_5/2_ respectively. It indicates that Bi exists in 3+ valence state in both samples^30^. Figure (c&g) shows the Fe 2p spectra of KBFO and 20N-KBFO samples respectively. The Fe 2p peak of KBFO splits into two peaks lying at 710.63 eV and 724.15 eV corresponding to Fe 2p_3/2_ and Fe 2p_1/2_ spin orbits respectively. For sample 20N-KBFO, Fe 2p_3/2_ and Fe 2p_1/2_ peaks lie at 709.75 eV and 723.15 eV respectively. Corresponding binding energies of Fe 2p_3/2_ and Fe 2p_1/2_ peaks indicate the existence of Fe^3+^ oxidation state in both the samples^30,34^. The peaks appearing above Fe 2p_3/2_ and Fe 2p_1/2_ peaks correspond to satellite peaks^35^. The slight shift of Fe 2p peak towards lower binding energy in sample 20N-KBFO attributed to the incorporation of N atoms in KBFO, is due to the lower electronegativity of N^34^. The XPS spectrum of O 1s is shown in Fig. 3d&h. The O 1s peak of KBFO could be fitted to two peaks at 528.9 eV and 530.72 eV, whereas the peaks for sample 20N-KBFO, lie at 529.43 eV and 531.62 eV respectively. The lower and higher binding energy peaks correspond to lattice oxygen and surface chemisorbed oxygen species respectively^34^. Figure 3i shows the presence of N 1 s peak in 20N-KBFO at 399.6 eV and is attributed to the presence of substitutional N in the form of Fe-(N–O) bonding. This implies that the lattice oxygen was partially substituted by N atoms^49,50^.

**Figure 4:**
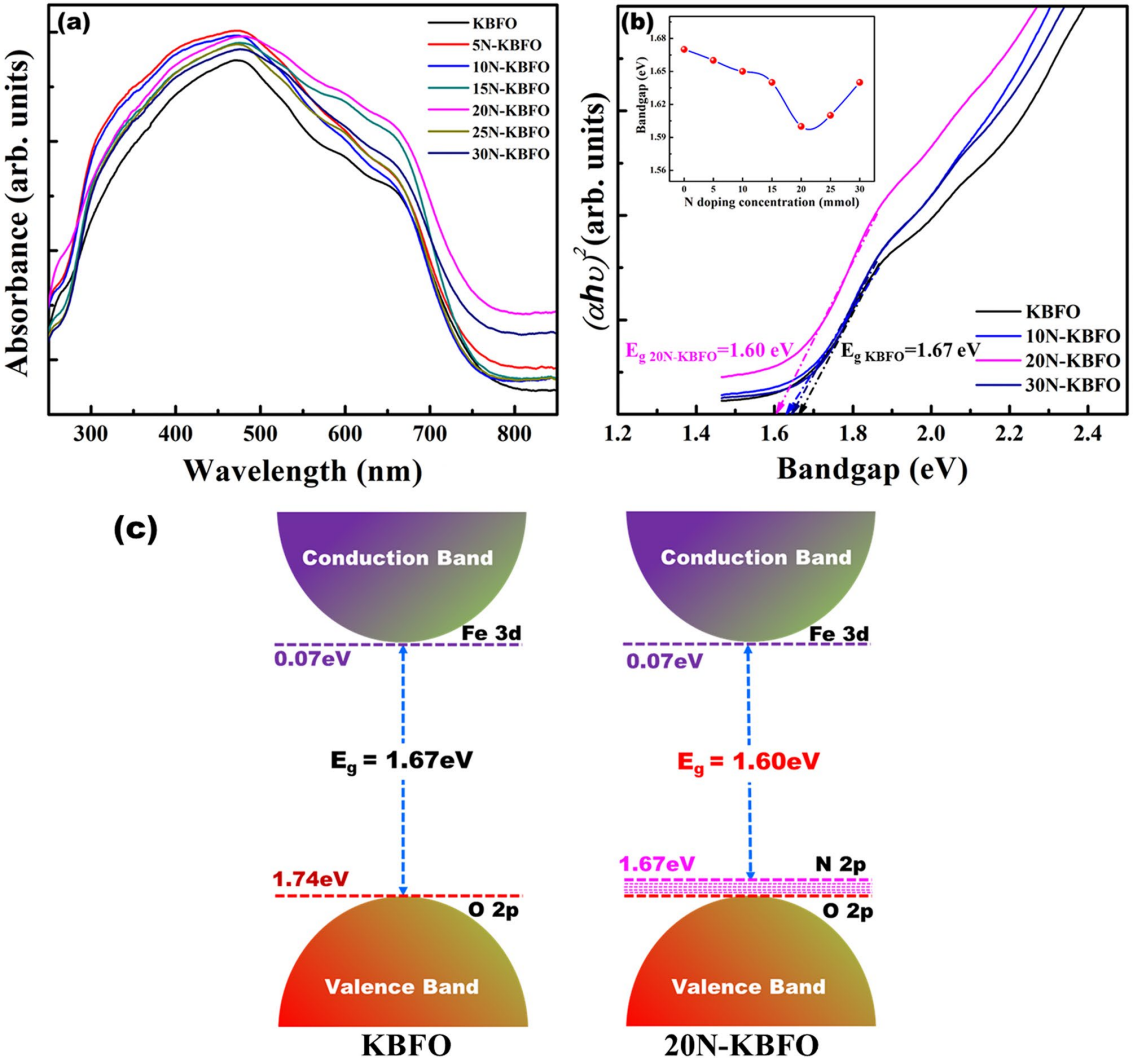
(**a**) UV–vis absorption spectra of KBFO with various N-doping concentrations (**b**) Tauc plots corresponding to KBFO, 10N-KBFO, 20N-KBFO, 30N-KBFO (inset shows variation of effective optical bandgap with respect to N-doping concentration) (**c**) Energy band diagram corresponding to pure KBFO and 20N-KBFO.

Figure 4(a) shows the UV–Visible absorption spectra of pure KBFO and N-doped KBFO with various doping concentrations. The absorption spectra of KBFO was broadened to NIR region after N-doping. The corresponding Tauc plots are shown in Fig. 4b. The effective optical bandgap of KBFO reduced from 1.67 to 1.60 eV (20N-KBFO) [Fig. 4b(inset)]. This reduction upon N-doping was attributed to the occupation of discrete midgap states of N 2p over O 2p states in valence band and also confirms the successful substitution of N atoms in O-sites^34,51^. A similar trend has been observed in previous reports^34^. Lower bandgap values are one of the important criteria for achieving enhanced visible light active photocatalysis. The energy band structures of KBFO and N-doped KBFO (Fig. 4c) were determined from Mulliken electronegativity expressions (Eqs. 3 and 4).

3 $$E_{CB} = \chi - E_{C} - \frac{1}{2}E_{g}$$

4 $$E_{VB} = E_{CB} + E_{g}$$

where E_CB_ and E_VB_ are the conduction and valence band edge positions, $\chi$ and *E*_*C*_ are the absolute electronegativity of compound and energy of free electron on hydrogen scale (4.5 eV) respectively. *E*_*g*_ is the corresponding bandgap energy.

**Figure 5:**
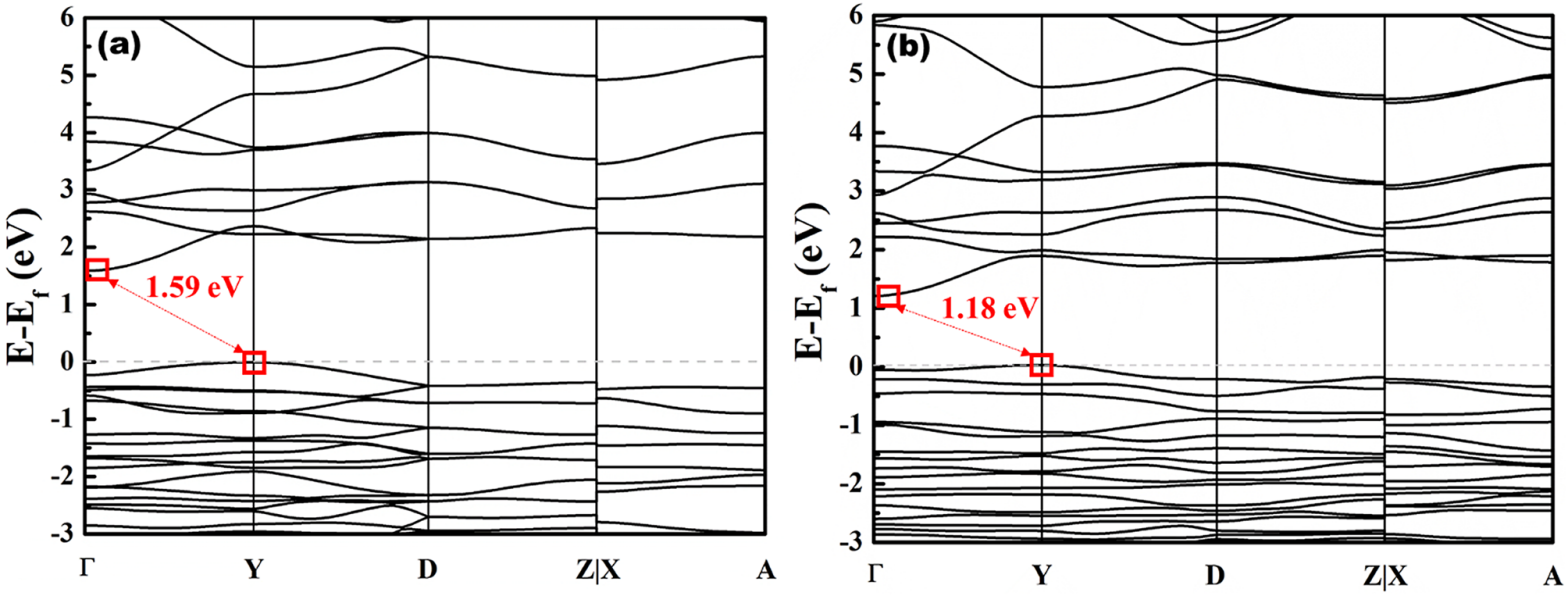
Electronic band structures of (**a**) KBFO and (**b**) N-KBFO calculated using DFT computations.

Theoretical bandgaps of KBFO and N-KBFO were calculated using density functional theory (DFT) calculations (Fig. 5a&b). DFT calculations were performed by sampling the Brillouin zone with a set of high symmetry k-points. The effect of Nitrogen doping in KBFO was analysed computationally and the bandgap of KBFO was found to be 1.59 eV. Upon replacement of few O atoms with N atoms in a unit cell of KBFO, the bandgap reduced to 1.18 eV, strongly supporting the experimental trend.

**Figure 6:**
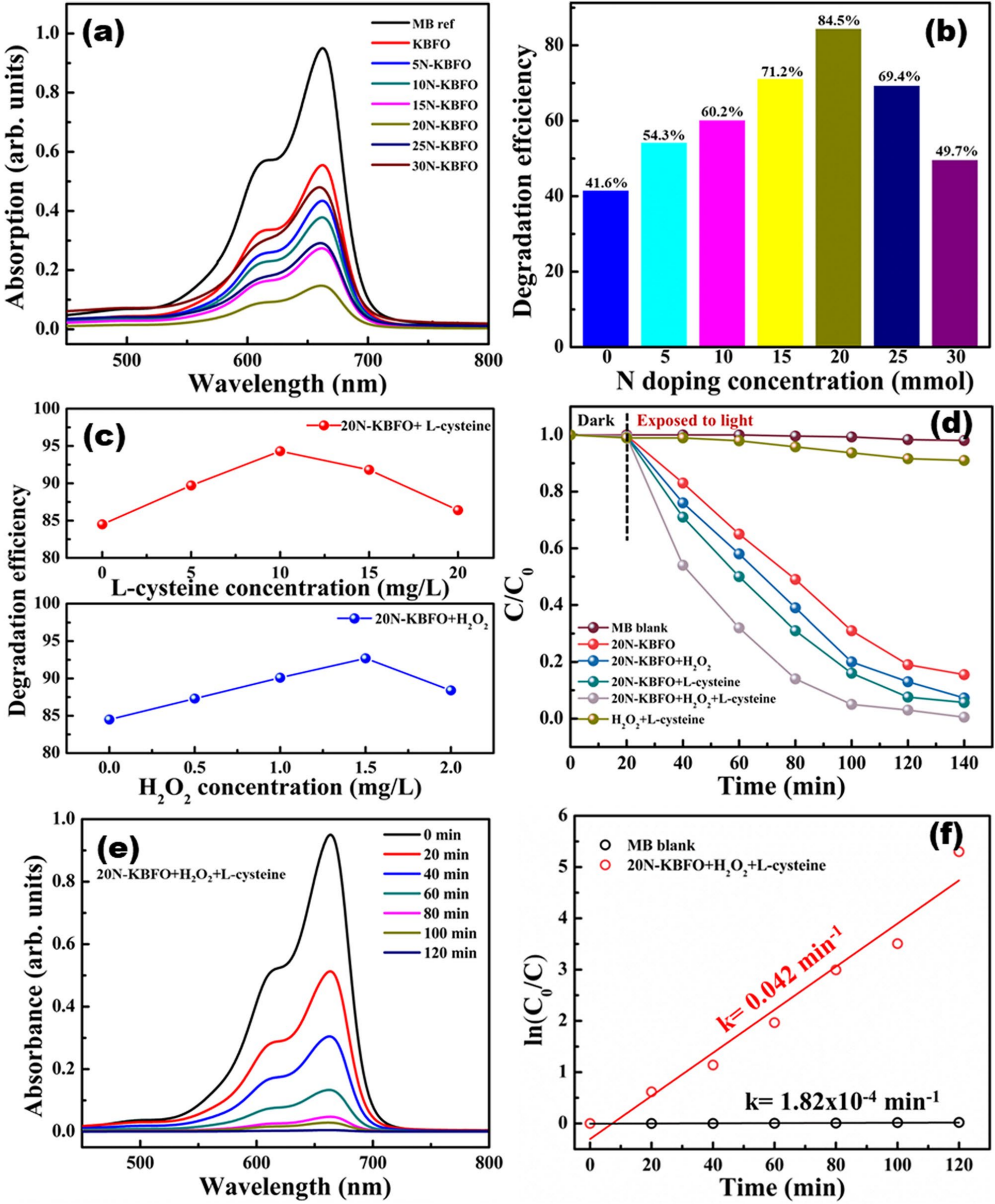
(**a**) Photocatalytic degradation profile of MB by KBFO with various N- doping concentrations. (**b**) Degradation efficacy chart of MB by KBFO with various N- doping concentrations (**c**) effects of H_2_O_2_ and L-cysteine concentration on the degradation of MB, (**d**) degradation (C/C_0_) of MB with H_2_O_2_ and L-cysteine systems. (**e**) Degradation profile of MB by 20N-KBFO + H_2_O_2_ + L-Cysteine. (**f**) first order reaction kinetics of MB by 20N-KBFO + H_2_O_2_ + L-Cysteine.

The catalytic activity of N doped KBFO samples were studied by degrading organic effluents MB and BPA as well as persistent antibiotics NOX and DOX. Photocatalytic degradation profile of MB by KBFO with various N- doping concentrations is shown in Fig. 6a. 20N-KBFO samples show better degradation efficiency (~ 84.5%), much higher than 41.6% for pure KBFO [Fig. 6(b)]. An increase in the photodegradation efficiency upon increasing N concentration in KBFO may be attributed to the narrow bandgap and efficient charge separation in N doped samples due to the presence of Fe–N active sites^34^. N-doping in KBFO shifts the absorption edge to enable it to absorb more sunlight as compared to bare KBFO. The modification of perovskite structures with transition metal-N active sites is desirable to enhance the charge transport features enabling higher catalytic activity towards remediation of wastewater. With an increase in N concentration over and above 20 mmol, the degradation efficiency starts decreasing and the results are consistent with optical absorption studies. Excess N incorporation induces defect levels in KBFO, which act as recombination centers, thus reducing the photodegradation efficiency^52^. Hence the optimum N incorporation was confined to 20 mmol.

The photocatalytic process is mainly governed by electron–hole (e^– _ ^h^+^) pairs generated in the catalyst upon light illumination and are responsible for redox reactions which mineralize the effluents. The photocatalytic mechanism of 20N-KBFO can be further enhanced by adopting fenton reactions with the addition of H_2_O_2_ in optimum quantity. Addition of H_2_O_2_ to an aqueous system containing an organic effluent and ferrous (Fe^3+^/Fe^2+^) ions lead to occurrence of complex redox reactions. The hydroxyl radicals and superoxide radicles generated in this process attach with the complex organic molecule and mineralize into nontoxic byproducts. The reversible redox reactions generate Fe^3+^/Fe^2+^ions and these reactions take place until effluents degrade completely. Recent studies have revealed that in addition to chelating agents like sulfur containing amino acid, L-cysteine improves the photo-fenton activity which allows generation of •OH active species by reacting with O_2_ and thus improve the catalytic performance. The optimization of dosage of fenton reagents (H_2_O_2_ and L-Cysteine) in photocatalysis enhances the performance as well as economic feasibility. In this work, H_2_O_2_ and L-cysteine dosage was optimized and found to be 1.5 mg/L and 10 mg/ml respectively. Upon addition of H_2_O_2_ the degradation efficiency of 20N-KBFO improved from 84.5 to 92.7% while with L-cysteine it improved to 94.7% [Fig. 6c]. The photo-fenton performance was also tested through different combinations of fenton reagents as shown in Fig. 6(d). MB almost degraded completely (99.5% with a rate constant about 0.042 min^-1^) post addition of both H_2_O_2_ and L-cysteine, which is only 9% for H_2_O_2_ + L-cysteine without any catalyst. These investigations imply that 20N-KBFO + H_2_O_2_ + L-cysteine combination is the best system for photo-fenton reaction for degrading MB. The degradation profile and first order reaction kinetics plot are shown in Fig. 6 (e & f).

**Figure 7:**
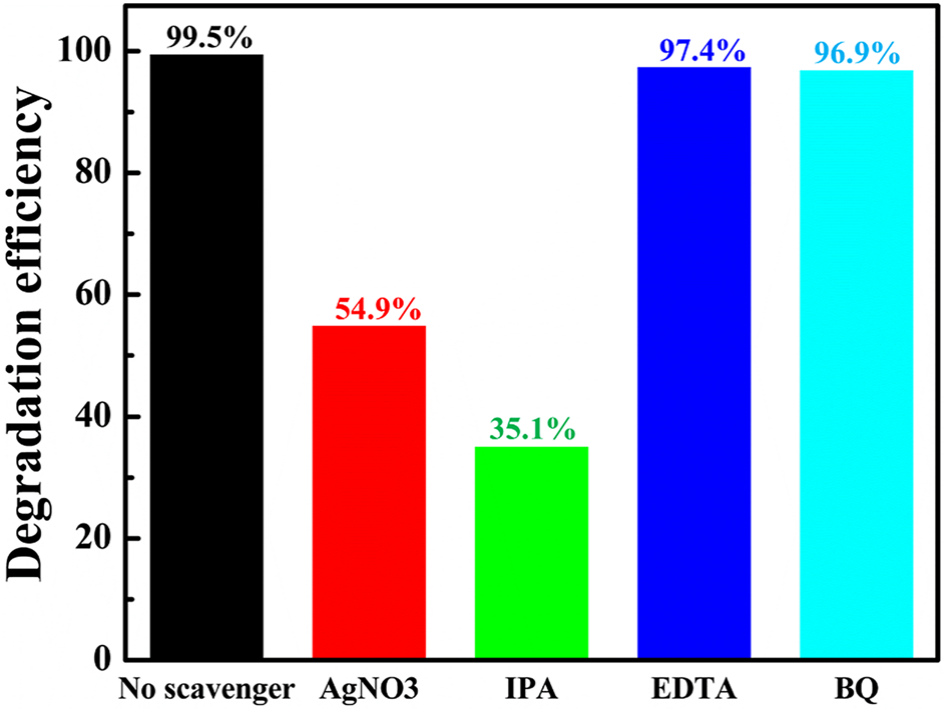
Effect of various of scavengers on the degradation of MB by 20N-KBFO + H_2_O_2_ + L-Cysteine.

**Figure 8:**
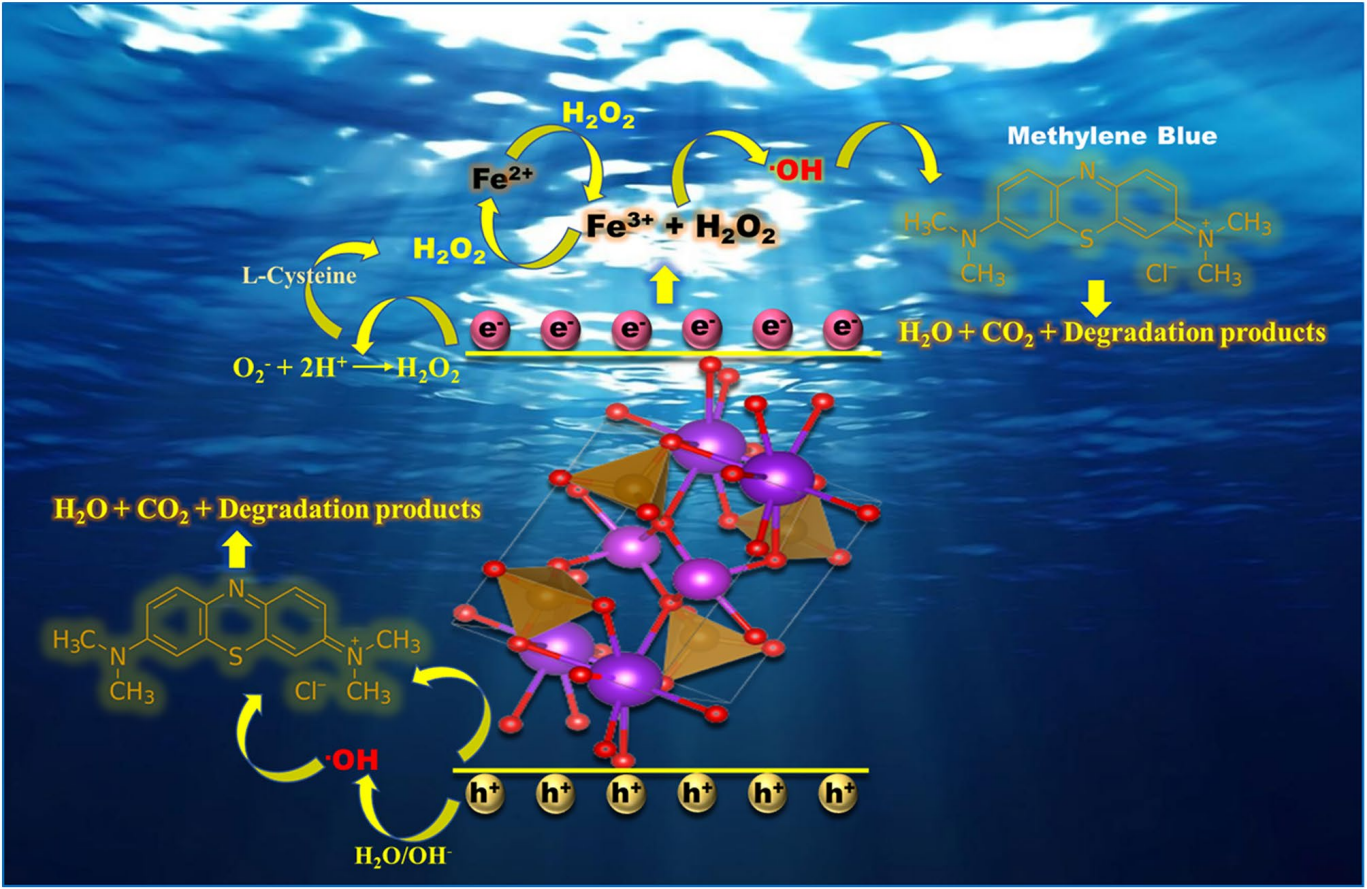
Photo-fenton degradation mechanism of MB using 20N-KBFO + H_2_O_2_ + L-Cysteine.

**Figure 9:**
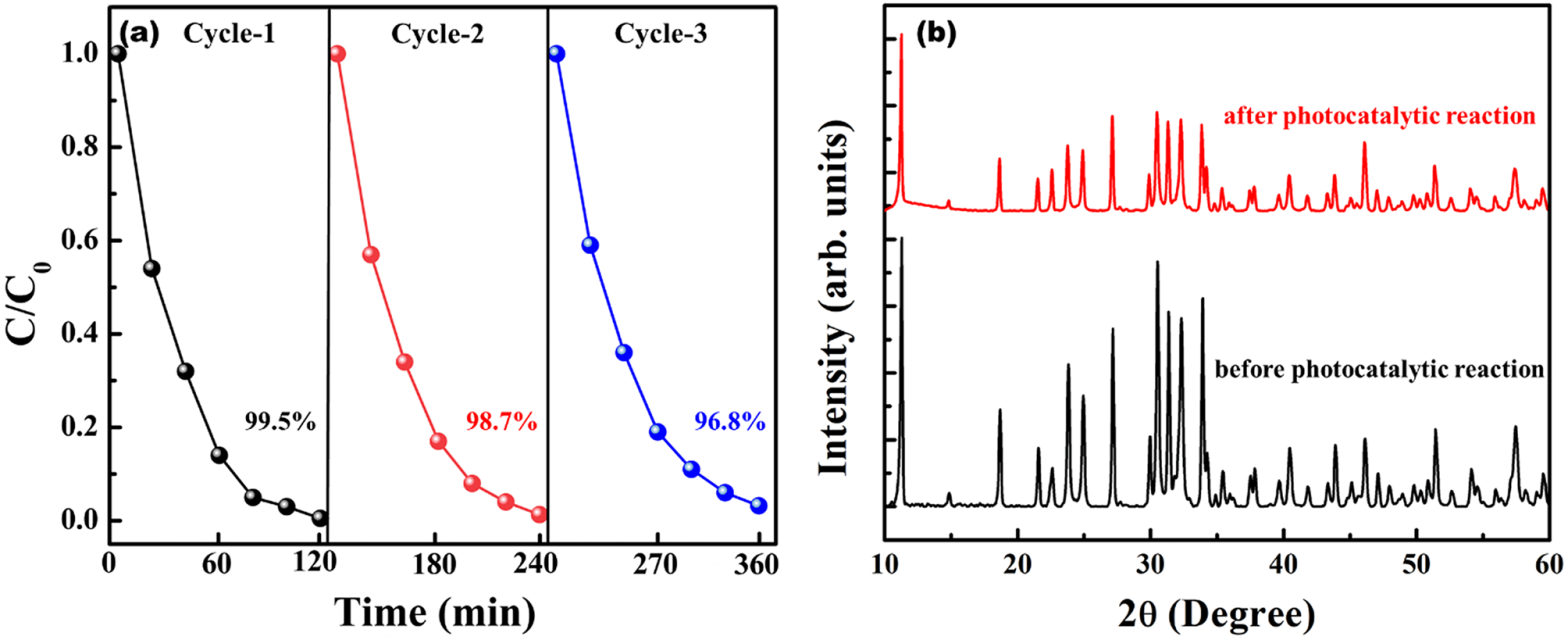
Reusability of 20N-KBFO for degradation of MB for three cycles (**b**) XRD pattern of 20 N-KBFO before and after three cycles of photocatalytic reaction.

In order to examine the active species involved in photo-fenton reaction, active species trapping experiments were conducted using various scavengers such as AgNO_3_, ethylenediaminetetraacetic acid (EDTA), isopropyl alcohol (IPA) and benzoquinone (BQ) and shown in Fig. 7. Sample 20N-KBFO + H_2_O_2_ + L-cysteine showed a photodegradation efficiency of about 99.5% without any scavenger. When AgNO_3_ (1 mmol) and IPA (1 mmol) were added to dye-catalyst suspension as e^-^ and •OH radical trapping agents, the photodegradation efficiency rapidly decreased to 54.9% and 35.1% respectively. The results point towards the role of e^-^ and •OH radicals being the main species responsible for photo-fenton mechanism. Upon addition of EDTA (1 mmol) and BQ (1 mmol) as h^+^ and superoxide (O_2_^.^) radicle trapping agents respectively, the degradation profile doesn’t change much. It implies that the role of h^+^ and superoxide (O_2_^.^) radicals in photo-fenton mechanism is negligible. The photo-fenton mechanism is thus mainly governed by e^-^ and •OH radicals. The major contribution of •OH radicals in this mechanism is due to addition of H_2_O_2_ and L-cysteine. A plausible degradation mechanism is illustrated in Fig. 8. The recyclability and stability of 20N-KBFO sample was investigated for three cycles. In all the three cycles, photodegradation performance of 20N-KBFO is negligible (Fig. 9a). The XRD pattern (Fig. 9) of recycled 20N-KBFO reveal that there are no structural transformations and secondary phases post three cycles of usage, stressing on the fact that the as prepared samples are reusable and stable for photocatalytic degradation of organic effluents.

**Figure 10:**
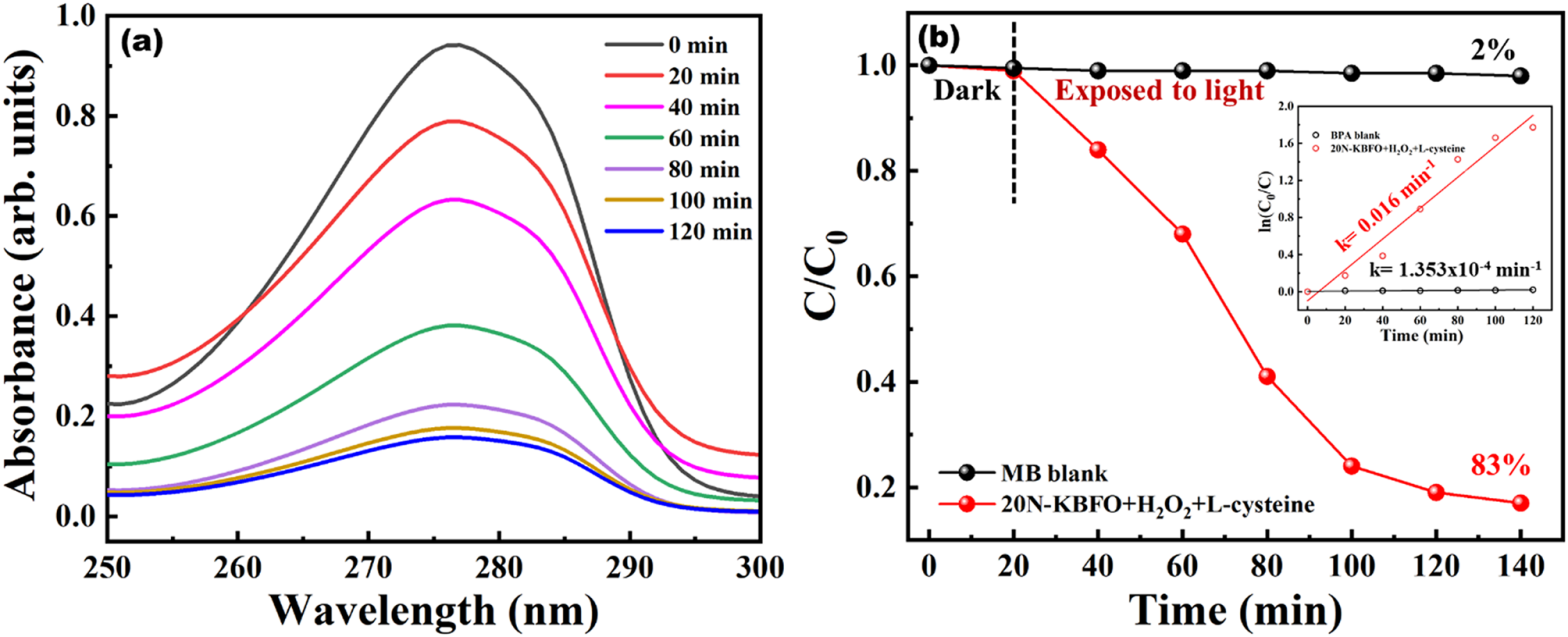
(**a**) Degradation profile of BPA by 20N-KBFO + H_2_O_2_ + L-Cysteine. (**b**) photocatalytic degradation (C/C_0_) of BPA by 20N-KBFO + H_2_O_2_ + L-Cysteine (inset showsfirst order reaction kinetics).

20N-KBFO + H_2_O_2_ + L-cysteine combination was further used to degrade the organic synthetic compound Bisphenol-A (BPA) under visible light. After exposing BPA-catalyst suspension in visible light for 120 min, BPA could be degraded upto 83% of its initial concentration with a rate constant of k = 0.016 min^-1^ whereas BPA alone degraded upto 2% only. The degradation profile and C/C_0 _plot ratio plots are shown in Fig. 10a,b.

**Figure 11:**
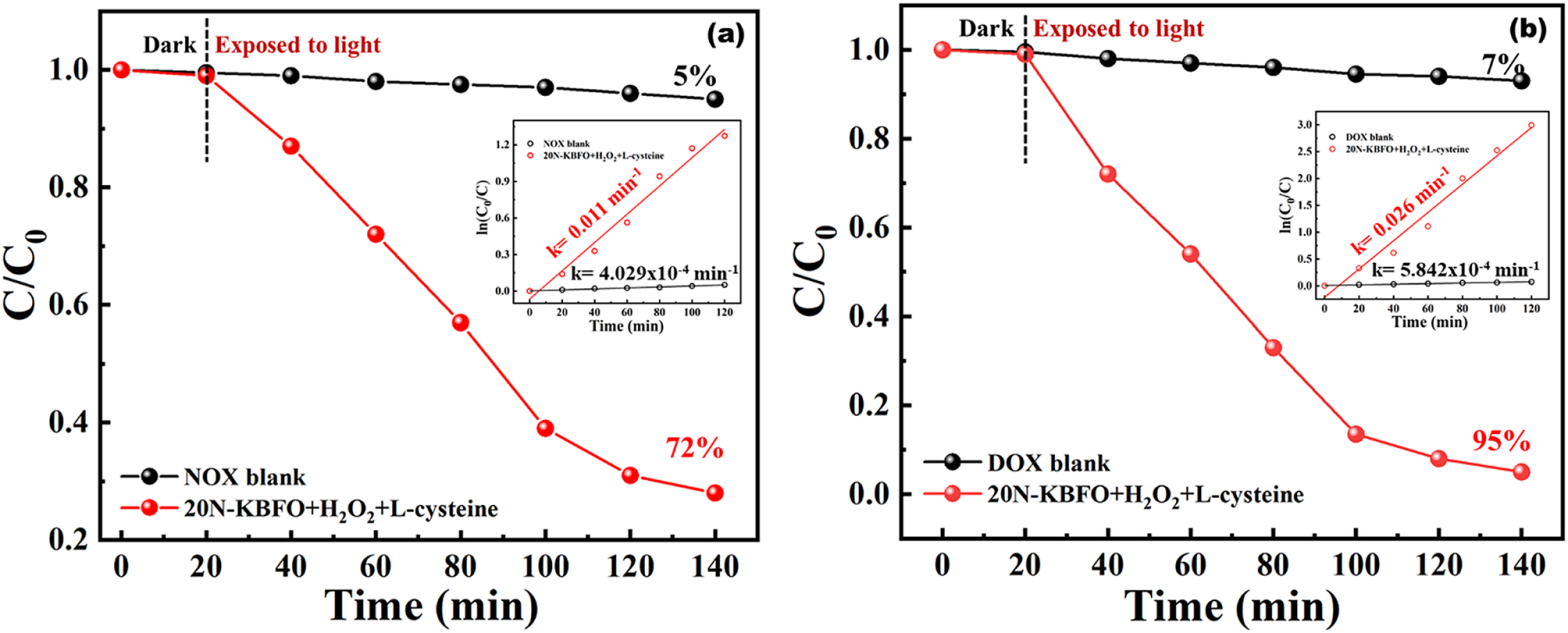
Photocatalytic degradation (C/C_0_) of (**a**) Norfloxacin (NOX) and (**b**)Doxycycline (DOX) by 20N-KBFO + H_2_O_2_ + L-Cysteine (inset first order reaction kinetics).

20N-KBFO + H_2_O_2_ + L-cysteine was also used for degrading antibacterial effluents such as NOX and DOX under visible light. NOX and DOX degraded by 72% (k = 0.011 min^-1^) and 95% (0.026 min^-1^) of its initial concentration. The degradation profile and C/C_0_ ratio plots are shown in Fig. 11a,b. These photo-fenton reaction studies with N-doped KBFO is a potential candidate for treating various effluents under sunlight.

**Figure 12:**
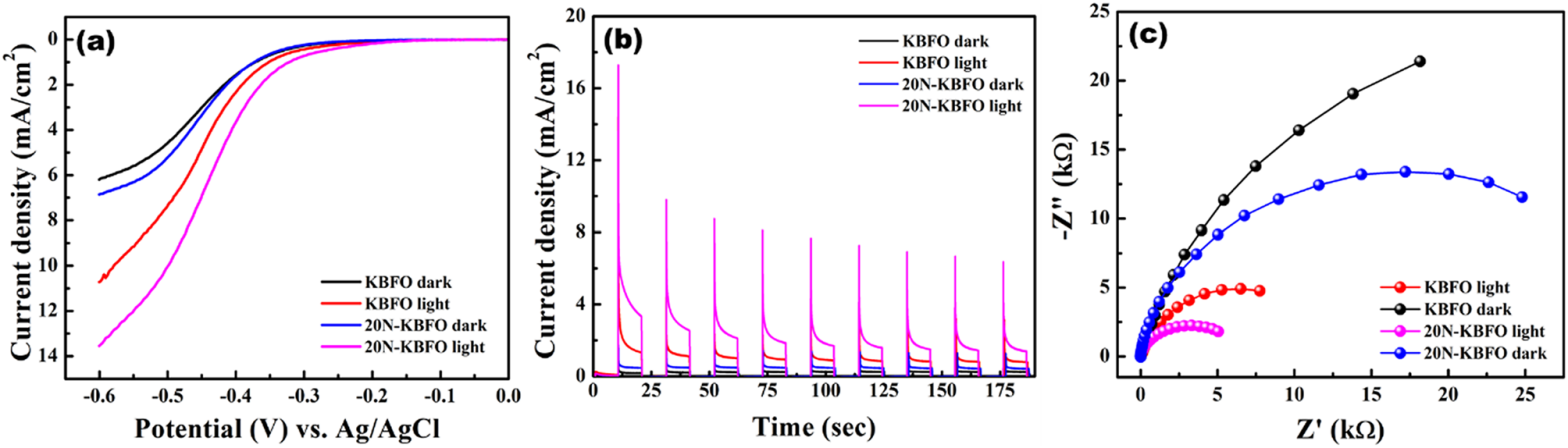
(**a**) I-V characteristics of KBFO and 20N-KBFO in dark and light illumination from LSV (0.6 V) (**b**) chronoamperometry curves of KBFO and 20N-KBFO in dark and light illumination (**c**) Photoelectrochemical impedance spectra of KBFO and 20N-KBFO in dark and light illumination.

The photoactivity of KBFO and 20N-KBFO were investigated and compared by photoelectrochemical (PEC) studies in 1 M Na_2_SO_4_ aqueous electrolyte solution. Linear sweep voltammetry (LSV), Chronoamperometry (CA) and electrochemical impedance spectroscopic (EIS) studies were carried out under dark and light illumination. Figure 12a shows the linear sweep voltammogram under dark and light for KBFO and 20N-KBFO exhibiting an enhanced photoresponse in 20N-KBFO over pure KBFO. The photocurrents corresponding to pure KBFO and 20N-KBFO were observed from CA studies at a potential of 0.6 V [Fig. 12b]. The average photocurrent density for KBFO was observed around ~ 4.31 mA/cm^2^under constant light illumination. For nitrogen doped KBFO (20N-KBFO) electrode, it increased to ~ 8.83 mA/cm^2^. The photocurrent density improved by two times in 20N-KBFO. Nyquist plots (Fig. 12(c) recorded using EIS shows the improved conductivity in nitrogen incorporated KBFO over KBFO. The samples show a rapid decrease in impedance under light illumination implying an efficient and rapid separation of photogenerated charge carriers under light irradiation leading to enhanced photoconductivity in 20N-KBFO^40^. The PEC studies revealed good photo response as well as efficient charge separation features in nitrogen doped KBFO over pure KBFO. The enhanced photocatalytic and photoelectrochemical properties of nitrogen doped brownmillerite KBFO make it a promising material for energy and environmental applications.

## Conclusion

Nitrogen doped KBiFe_2_O_5_ was successfully synthesised using melamine (C_3_H_6_N_6_) as the N source. Systematic investigations on structural, morphology and optical properties of as prepared samples were carried out. Optimum nitrogen incorporation in KBFO was analysed by degrading MB and 20 mmol of N doped KBFO was found to be the best sample for photo-fenton activity. Combination of H_2_O_2_ + L-cystyein was used as fenton reagent and the photo-fenton activity in presence of 20N-KBFO + H_2_O_2_ + L-cysteine showed rapid improvement in photodegradation efficiency by generating more active species like •OH (as confirmed from active species trapping experiments). Reusability and stability studies were performed upto three cycles and the samples show stable catalytic performance without any structural change. The performance of 20N-KBFO, LSV, CA and EIS studies revealed an enhanced photoresponse in 20N-KBFO over pure KBFO. Lower bandgap, high photodegradation efficiency, stability and satisfactory photoresponse exhibited by N-doped KBiFe_2_O_5_ make it one of the best brownmillerite compound for energy and environmental applications.
